# Recent Advances in Site-Specific Lipid Nanoparticles for mRNA Delivery

**DOI:** 10.1021/acsnanoscienceau.2c00062

**Published:** 2023-03-30

**Authors:** Xiao Xu, Tian Xia

**Affiliations:** † Division of NanoMedicine, Department of Medicine, University of California, Los Angeles, California 90095, United States; ‡ California NanoSystems Institute, University of California, Los Angeles, California 90095, United States

**Keywords:** nanotherapeutics, mRNA, lipid nanoparticles, vaccine, targeted delivery, organ-specific, cell-specific, off-target effects

## Abstract

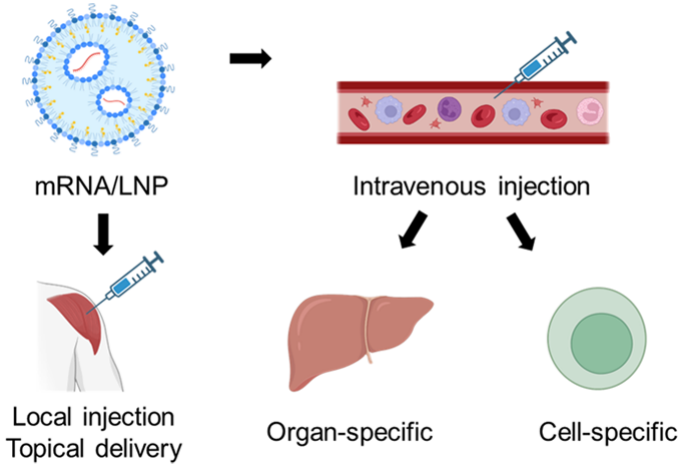

The success of mRNA vaccines during the COVID-19 pandemic has greatly accelerated the development of mRNA therapy. mRNA is a negatively charged nucleic acid that serves as a template for protein synthesis in the ribosome. Despite its utility, the instability of mRNA requires suitable carriers for *in vivo* delivery. Lipid nanoparticles (LNPs) are employed to protect mRNA from degradation and enhance its intracellular delivery. To further optimize the therapeutic efficacy of mRNA, site-specific LNPs have been developed. Through local or systemic administration, these site-specific LNPs can accumulate in specific organs, tissues, or cells, allowing for the intracellular delivery of mRNA to specific cells and enabling the exertion of local or systemic therapeutic effects. This not only improves the efficiency of mRNA therapy but also reduces off-target adverse effects. In this review, we summarize recent site-specific mRNA delivery strategies, including different organ- or tissue-specific LNP after local injection, and organ-specific or cell-specific LNP after intravenous injection. We also provide an outlook on the prospects of mRNA therapy.

## 1 Introduction

Nucleic acid therapy is an emerging therapy in recent years, which could be used to treat many diseases that cannot be targeted by small molecules.^1,2^ Four main types of nucleic acid therapeutics has been approved by FDA, including antisense oligonucleotides (ASOs), ligand-modified short interfering RNA (siRNA) conjugates (e.g., N-acetylgalactosamine (GalNAc)), lipid nanoparticles (LNPs), and adeno-associated virus (AAV) vectors.^3^ The purpose of these nucleic acid therapies is to up- or down-regulate the expression of specific proteins.^4^ For example, siRNA is used to silence paired mRNA to inhibit protein generation and mRNA is used as a template to produce specific proteins to exert their functions.^5^

**Figure 1:**
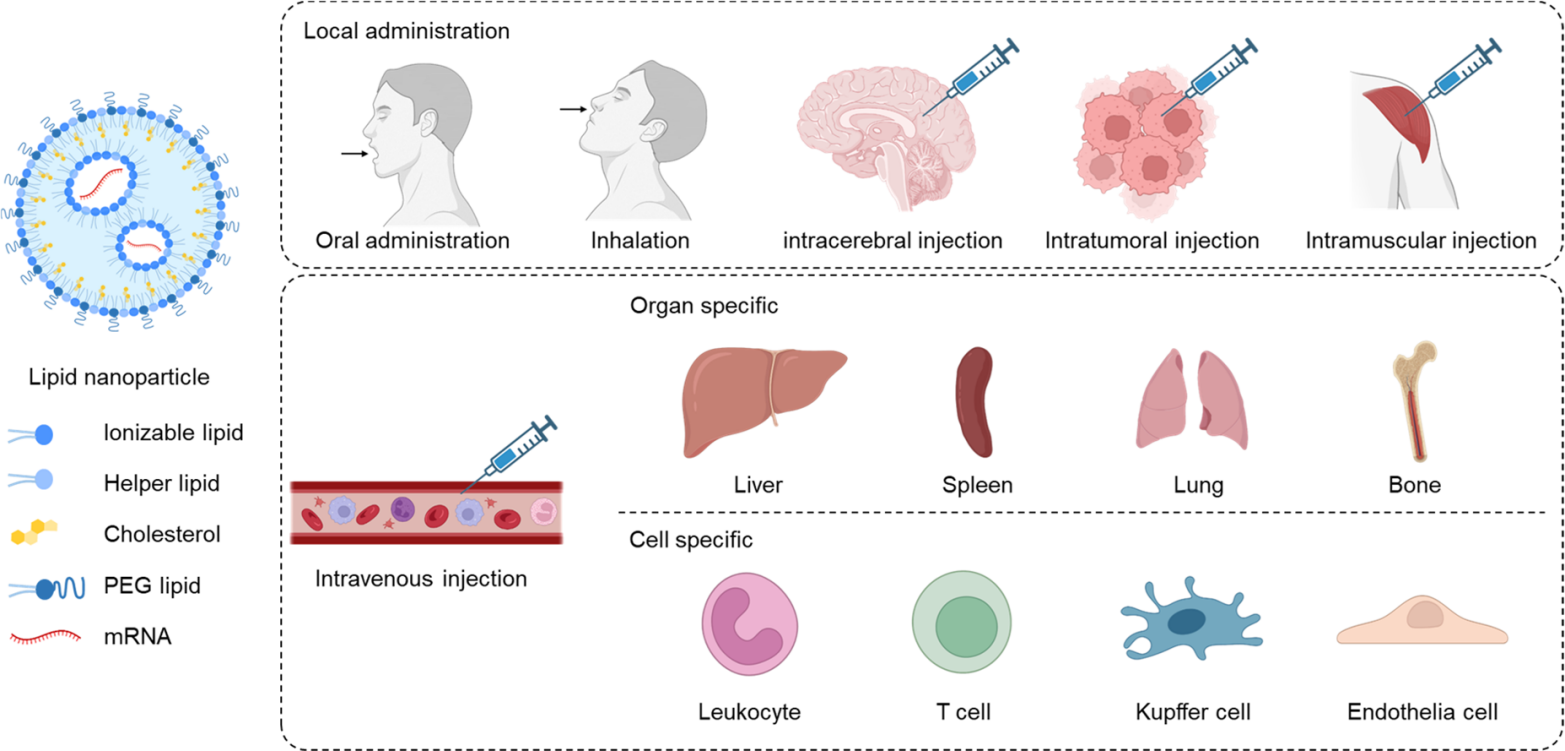
Site-specific lipid nanoparticles (LNP) for mRNA delivery. mRNA-loaded LNP is composed of ionizable lipid, helper lipid, cholesterol, and PEG lipid. LNP could be administered locally or systemically, to realize the organ, tissue, or cell-specific mRNA delivery.

Among these four types of nucleic acid therapies, LNP is most attractive because it can be used to deliver many kinds of nucleic acids without changing their structure. The type of nucleic acid that can be encapsulated in LNP includes but is not limited to mRNA, microRNA (miRNA), siRNA, and single guide RNA (sgRNA).^6^ LNP is composed of different lipids, which makes it easier for quality control since lipids are easier to purify than other types of carriers, such as macromolecules or viruses.^7^ Furthermore, it is easy to develop new LNP by changing the lipid structures or lipid compositions, which added the versatility of LNP.^8^ Generally, LNP was composed of four types of lipids, including ionizable lipids, helper lipids, cholesterol, and PEGylated lipids (Figure 1). The ionizable lipid is the most important component that is responsible for nucleic acid encapsulation in preparation and lysosomal escape after cellular uptake.^9^ The headgroup of ionizable lipids is positively charged at acidic conditions, which allows the electrostatic interaction with the negatively charged nucleic acid and increases the encapsulation efficiency.^10^ The hydrophobic alkyl chains of ionizable lipids are unsaturated, which form a hexagonal lipid phase and enhance the endosome escape of mRNA from LNP after they are taken up by cells.^11^ In addition to ionizable lipids, the other three components are also important to LNP. Helper lipids and cholesterol increase the stability of LNP and enhance the endocytosis by cells. PEGylated lipids are important for increasing the stability and prolonging the circulation time of LNPs, which improves delivery efficiency after intravenous injection.^12^

Significant efforts have been dedicated to developing LNP, which has resulted in the approval of the first FDA-approved drug, Onpattro (Patisiran), in 2019. This siRNA drug utilizes LNP for the treatment of polyneuropathy resulting from hereditary transthyretin-mediated amyloidosis (hATTR amyloidosis).^13^ The ionizable lipid used in Onpattro is DLin-MC3-DMA, which comprises 50% of the total lipid composition. Upon intravenous injection, this LNP can be successfully delivered to the liver, with research indicating that this targeting effect is attributed to the dissociation of polyethylene glycol (PEG) from LNP and the nonspecific adsorption of apolipoprotein E (ApoE) in the circulation. ApoE functions as a ligand for low-density lipoprotein receptors (LDLR), which are predominantly expressed by hepatocytes, thereby explaining why LNP accumulates in the liver.^1,14−16^ Studies have demonstrated that the siRNA employed in the delivery system can silence transthyretin (TTR) mRNA and alleviate hATTR symptoms.

The pandemic of COVID-19 accelerates the development of LNP-based mRNA vaccines, and two of them were approved in 2021, which are mRNA-1273 (Moderna) and BNT162b2 (Pfizer).^17,18^ Both of them activate the immune system to resist viral infection after intramuscular injection. The ionizable lipids used in these formulations are SM-102 and ALC-0315, and the mRNA used in these LNP encodes the spike (S) proteins. The S protein is responsible for binding to the human angiotensin-converting enzyme-2 (ACE2) receptor, initiating cellular uptake by the lung alveolar epithelial cells.^19^ After local injection, the LNP will be uptake by somatic cells, followed by mRNA release and translation. The expressed S proteins are taken up by local antigen-presenting cells (APCs), which subsequently present them to T cells to initiate the immune response and generate specific antibodies against SARS-CoV-2. LNP can also carry two or more different nucleic acids to perform gene editing in cells.^20^ The combination of Cas9 mRNA (mRNA) and single-guide RNA (sgRNA) is the commonly used pair for CRISPR-Cas9 gene editing. Extensive research has been dedicated to developing a safe and highly efficient gene editing tool in this field.

Although the LNP-based mRNA drug delivery system has shown promising results, there is still significant room for improvement. The primary focus is on enhancing delivery efficiency while reducing unwanted side effects. For instance, some individuals experience varying degrees of side effects, such as injection site pain, fever, fatigue, headache, and diarrhea, following one or two doses of the COVID-19 mRNA vaccine.^21^ Although some side effects are associated with immune responses, efforts to reduce the incidence of adverse effects remain crucial.

There is ongoing research on improving the precision of mRNA-loaded LNP delivery to specific cells, tissues, or organs, which could enhance efficacy and reduce side effects. This review summarizes recent studies utilizing site-specific LNP for mRNA delivery, in order to expand the application of mRNA therapy with high efficiency and low systemic toxicity (Table 1).

**Table 1:** Summary of Different Site-Specific LNP^a^

Administration route	Targeting site	LNP composition	mRNA	Disease	ref
Oral	Colon	phosphatidic acid, monogalactosyldiacylglycerol, and digalactosyldiacylglycerol	IL-22 mRNA	Inflammatory bowel disease	(26)
Inhalation	Lung	modified PEI compound 7C1, cholesterol, DMG-PEG_2000_, and cationic lipid DOTAP	mRNA encoded with broadly neutralizing antibody targeting haemagglutinin	H1N1 infection	(31)
Intramuscular injection	Muscle	TCL053, DPPC, PEG-DMG, Cholesterol	Cas9 mRNA and sgRNA	Duchenne muscular dystrophy	(32)
Intracerebral injection	Tumor	ionizable lipid, DSPC, cholesterol, DMG-PEG, and DSPE-PEG	Cas9 mRNA and sgPLK1	Orthotopic glioblastoma	(36)
Intratumor injection	T cells	PL1, DOPE, Cholesterol, DMGPEG_2000_	OX40 costimulatory receptor mRNA	melanoma	(42)
Intravenous injection (organ specific)	Liver	MC3 lipid, Cholesterol or β-sitosterol, DMG-PEG_2000_, DSPC	hsACE2 mRNA	SARS-CoV-2	(45)
	Liver	PEG lipid, Ionizable lipid, Structural lipid, Cholesterol	HNF4A mRNA	liver fibrosis and cirrhosis	(48)
	Liver	Ionizable lipid, Helper lipid, Cholesterol, PEG lipid	BisCCL2/5i mRNA	liver cancer	(53)
	Liver	cationic lipid-like molecule G0-C14, PDSA, PEG lipid	p53 mRNA	liver cancer	(59)
	Liver	5A2-SC8, DOPE, cholesterol, PEG lipid	Cas9 mRNA, PD-L1 sgRNA, and FAK siRNA	liver cancer	(62)
	Liver and spleen	ionizable lipid IC8, DSPC, cholesterol, DMG-PEG	B7H3-CD3 mRNA	hematologic malignancies and melanoma	(63)
	Liver	ionizable lipid, DOPE, cholesterol, and PEG lipid	Cas9 mRNA and mouse antithrombin targeted sgRNA	hemophilia	(69)
	Liver	Ionizable lipid, DSPC, cholesterol, PEG-DMG	hPBGD mRNA	acute intermittent porphyria	(71)
	Spleen, lung, or liver	SORT lipid, ionizable lipid, helper lipid, cholesterol, and DMG-PEG	Cas 9 mRNA and sgRNA	N.A.	(72)
	Bone	lipids with bisphosphonates head groups	BMP-2 mRNA	skeletal diseases	(76)
Intravenous injection (cell-specific)	Leukocyte	Anti-Ly6c mAbs, MC3, DSPC, Cholesterol, DMG-PEG, and DSPE-PEG	IL-10 mRNA	inflammatory bowel disease	(77)
	T cell	Anti-CD5 antibody, Ionizable lipid, phosphatidylcholine, cholesterol, and PEG lipid	FAP-CAR mRNA	cardiac fibrosis	(83)
	Kupffer cells and liver endothelial cells	Oxidized Cholesterol, cKK-E12, PEG lipids, DOPE	Cre mRNA	N.A.	(86)
	SECs/LSECs	MC3, DSPG, cholesterol, DMG-PEG_2000_	eGFP mRNA or mCherry	N.A.	(87)
	LSECs	MC3, DSPC, cholesterol, DSPE-PEG_2000_-Mannose	Ara h2 mRNA	Peanut allergy	(85)
	Kupffer cells and liver endothelial cells	ionizable lipid, DOPE, cholesterol, and PEG lipid	Luc mRNA and Cre mRNA	N.A.	(88)

a PL1: phospholipid derivatives 1, hsACE2: human angiotensin-converting enzyme 2, HNF4A: human hepatocyte nuclear factor alpha, FAK: focal adhesion kinase, BMP-2: bone morphogenetic protein-2, FAP: fibroblast activation protein, hPBGD: human porphobilinogen deaminase, N.A.: not applicable.

Improvement could be made to mRNA-loaded LNP to deliver the mRNAs to a specific cell, tissue, or organ more precisely, which will make it more effective with reduced side effects. Recently, many scientists are focusing on site-specific mRNA delivery systems. To design a better mRNA carrier, three things should be considered, including the targeted cell or tissue, the administration route, and the targeting strategies. This review highlights recent studies utilizing site-specific LNP for mRNA delivery, in order to broaden the application of mRNA therapy with high efficiency and low systemic toxicity. (Table 1)

## 2 Site-Specific LNP by Local Administration

The route of administration is critical for delivering mRNA-loaded LNP. Various administration routes have been exploited to achieve site-specific delivery of LNP, including oral administration, inhalation, and local injection (intramuscular, intratumoral, and intracerebral injection).

### 2.1 Oral Administration

Oral administration is a widely used, convenient and well-compiled route of administration.^22^ However, the harsh acidic environment and presence of enzymes in the gastrointestinal (GI) system may limit drug efficacy. Oral administration of mRNA poses significant challenges due to the susceptibility of the molecule to degradation by nucleases and the harsh acidic environment in the GI tract. Nonetheless, research is ongoing to develop LNPs that can be taken orally and interact directly with GI disease sites to improve drug efficacy.

Inflammatory bowel disease (IBD) is a hard-to-cure disease that affects many adults in the US.^23^ Chronic inflammation of the intestinal tract greatly affects the patient’s quality of life, and unfortunately, there is no drug available to treat the disease.^24^ Interleukin-22 (IL-22) is an important cytokine that promotes the proliferation of epithelial cells and maintains the homeostasis of epithelial cells. The IL-22-mediated signaling pathway enhances anti-inflammatory effects and promotes the regeneration of local tissue.^25^ Increasing the local level of IL-22 in IBD ulcers may reverse the inflammatory microenvironment and inhibit the progress of IBD. Based on this fact, Sung et al. prepared a LNP loaded with IL-22 encoded mRNA. The LNP was composed by phosphatidic acid, monogalactosyldiacylglycerol, and digalactosyldiacylglycerol at the molar ratio of 5:2:3, and the mRNA loaded LNP was with around 200 nm diameter and −18 mV of surface charge.^26^ Oral administration of IL-22 mRNA loaded LNP significantly increased the expression of IL-22 in the colonic mucosa and accelerated the healing of colitis in mouse models, evidenced by the recovery of body weight and colon length. These results demonstrated the oral administration of mRNA loaded LNP is a feasible strategy to treat intestinal diseases by reconstruction of intestinal microenvironment.

### 2.2 Inhalation

Inhalation is also a preferred drug administration route.^27^ Due to the large absorption area and rich pulmonary blood flow, the drugs inhaled can be quickly transferred to blood circulation, which increases the bioavailability of drugs. However, it is hard to control the dose by inhalation, and the clearance in the airway increases the difficulty. It is reported that the inhaled aerosols experience two types of clearance based on their size and deposition regions. Aerosols larger than 5 μm will be cleared by mucociliary clearance, and more than 80% of aerosols will be removed. In contrast, aerosols smaller than 5 μm undergo macrophage clearance.^28^ Nebulization is the most commonly seen method for inhalation.^29^ Since the main target of the COVID-19 virus is the lung, scientists are working on developing nebulized LNPs to treat infectious diseases. Although the advanced aerosolization techniques could help the drugs enter the lung, the shearing forces may disrupt the structure of nanoparticles and the physical barriers in the airways may make it hard to reach the target.^30^ To solve these problems, Dahlman and co-workers reported a screening method to identify the best LNP composition for mRNA delivery by nebulization.^31^ The results showed that a higher molar ratio of PEG lipids in LNP will improve the performance of cationic helper lipids, which is important for low-dose mRNA delivery by nebulization. They prepared an mRNA-loaded LNP for lung delivery, which was composed of modified PEI compound 7C1, cholesterol, DMG-PEG_2000_, and cationic lipid DOTAP. The high percentage of DMG-PEG_2000_ (55%) enhanced the lung delivery of LNP, which is better than clinically used LNP. Follow-up studies found that this LNP significantly protected mice against H1N1 influenza when mRNA encoded with a broadly neutralizing antibody targeting haemagglutinin was loaded.

### 2.3 Local Injection

Local injection means the drug is injected into a small area of the body. These drugs can not only affect the local site but can also diffuse or be transferred to blood circulation and exert a systemic therapeutic effect. COVID-19 vaccine is a typical example that works systemically after intramuscular injection. Currently, an increasing number of mRNA-based studies are focused on local injection, which can provide targeted therapy at the injection site, while minimizing the potential for systemic off-target effects. Kenjo et al. developed an LNP-based mRNA delivery system to treat Duchenne muscular dystrophy (DMD), which was caused by the loss-of-function mutation of the dystrophin gene.^32^ Although ASO drugs have been used to treat DMD patients by restoring the reading frame of the dystrophin protein, the transient therapeutic effects required repeated injection for a patient.^33^ The CRISPR-Cas9 system can be used to restore the dystrophin expression with long-lasting effect, while the proper delivery carrier is needed to deliver Cas9 mRNA and sgRNA into target cells.^34^ These researchers synthesized ionizable lipids with triple hydrophobic alkyl tails, which were then used as a component to formulate the LNP vesicle to deliver Cas9 mRNA and specific sgRNA. This LNP showed great local therapeutic effect after intramuscular injection, and systemic therapeutic effect after limb perfusion in the DMD mouse model represents a promising carrier for delivering CRISPR-Cas9 gene editing tool.

Local injection of LNP-formulated CRISPR-Cas9 gene editing tool could also be used for cancer treatment after local injection.^35^ Recently, Dan Peer and co-workers developed a CRISPR-LNP for the treatment of glioblastoma and ovarian malignancy.^36^ PLK1 is an essential kinase for mitosis, which is highly expressed in many tumor cells.^37^ Targeted edit of *PLK1* gene is a promising method to induce tumor cell apoptosis.^38^ To maximize the delivery efficiency of CRISPR LNP, they screened an ionizable amino lipid library and did a series of *in vitro* experiments. They found that L8 is the best ionizable cationic lipid for CRISPR LNP, instead of the MC3 lipid for siRNA delivery. Then, the Cas9 mRNA and sgRNA for PLK1 (sgPLK1) were coloaded into the LNP and intracerebral injected into the mouse with orthotopic glioblastoma. This CRISPR LNP significantly inhibited tumor growth by 50% and improved the survival rate by 30%, representing a good mRNA drug for cancer treatment. Furthermore, they studied if surface modification of LNP could increase selective uptake by tumor cells. The CRISPR LNP was modified with anti-EGFR antibody and intraperitoneally injected into a disseminated ovarian tumor bearing mouse. Antibody-modified LNP could increase the expression of mRNA loaded proteins in target tumor cells by three times and significantly increase survival rate to 80%. Taken together, the local injection of CRISPR LNP (Cas9 mRNA and sgPLK1) could specifically induce the tumor regression.

**Figure 2:**
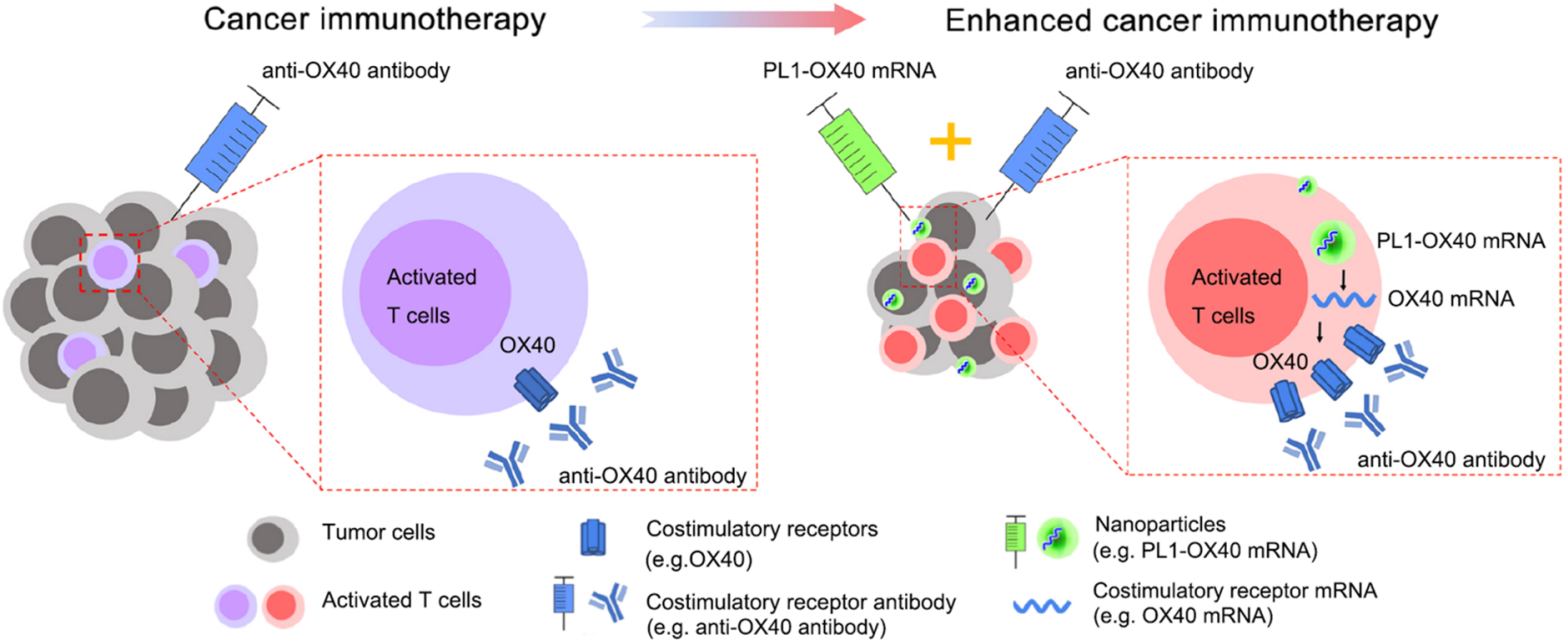
Intratumor injection of LNP loaded with OX-40 mRNA for enhanced cancer immunotherapy. Anti-OX40 antibody was proven to be effective in augmenting antitumor immunity in many kinds of cancers, which specifically bind with the costimulatory receptor OX40 on T cells and induce T cell immunity. To enhance the T cell response, LNP loaded with OX-40 mRNA was injected intratumorally, which induced the increased expression of OX40 receptors on T cells and burst the immunotherapy efficacy of the anti-OX40 antibody. Reprinted with permission under a Creative Commons CC BY License from ref (42). Copyright 2021 Springer Nature.

Intratumor injection of mRNA drugs can also enhance the local immune response, which was accomplished by expressing costimulatory receptors on tumor-infiltrating T cells. The interactions between costimulatory molecules and receptors activate the proliferation and directed differentiation of T cells, which is important for cancer therapy.^39^ OX40 (CD134) is a T-cell costimulatory receptor, and the activation of the OX40 signaling pathway will boost the antitumor immune response.^40,41^ To realize the constant expression of OX40 receptors in tumor-infiltrating T cells, the mRNA encoded with OX40 costimulatory receptor proteins were loaded into phospholipid nanoparticles (PL1), which were then injected into tumors (Figure 2).^42^ The combination of PL1-loaded OX40 mRNA and anti-OX40 antibody therapy boosts the immune response in the A20 tumor-bearing mouse model, resulting in a 60% complete response. Moreover, this treatment could also increase the therapeutic effect of anti-PD-1 and anti-CTLA-4 antibodies, which may arise from the activation of multiple immune cells in the tumor microenvironment.

## 3 Organ-Specific LNP by Intravenous Administration

Intravenous injection is another standard route of administration, with a bioavailability of 100%.^43^ The biodistribution of LNP after intravenous injection is very important since off-target delivery of mRNA may lead to adverse reactions and greatly reduce therapeutic efficacy. Recently, many research works were focused on organ-specific LNP It is reported that the biodistribution of LNP will be greatly influenced by their physical properties, including particle size, shape, surface charge, and structure.^44^ These physical properties are mainly determined by the lipid structure and lipid compositions. That is why efforts are focused on using new lipids or optimizing lipid compositions for organ-specific LNP.

### 3.1 Liver-Targeted LNP

The marketed Onpattro siRNA-loaded LNP mainly accumulates in the liver after intravenous injection. The targeting mechanism is achieved by the use of a 14-carbon lipid of DMG-PEG_2000_ in the LNP formulation, which quickly dissociates from the LNP surface in the circulation due to weak association with the surface by the short 14-carbon chains. After that, ApoE binds to the LNP to form a corona, which is recognized by LDLR on hepatocytes and promotes the endocytosis of LNP.^14^ A lot of studies followed the prescription of Onpattro to prepare the liver-targeted LNPs. These mRNA-loaded LNP deliver specific mRNA to the liver to treat other types of liver-related diseases, including infectious disease, liver fibrosis, liver cancer, hereditary diseases, etc.

Sahay’s group recently developed a liver-targeting LNP to deliver mRNA for SARS-CoV-2.^45^ The mRNA encodes a soluble form of human angiotensin-converting enzyme 2 (hsACE2), which is the target for SARS-CoV-2 spike protein and mediates the endocytosis of SARS-CoV-2 into human airway cells.^46,47^ The LNP composition was same to the formulation of Onpattro. After intravenous injection, this mRNA-loaded LNP accumulates in the liver and produces the hsACE2. The production of hsACE2 will continue for several days. Unlike virus-based delivery system, LNP can be repeatedly injected to maintain the hsACE2 above effective concentration. This research work proved that hsACE2 mRNA therapy could be used to prevent SARS-CoV-2 infection, which expands the choice of vaccines for defending against COVID-19.

mRNA-loaded LNP could also be used to treat liver fibrosis and cirrhosis. Yang et al. developed an mRNA-loaded LNP to deliver mRNA to the liver.^48^ The protein encoded by mRNA was human hepatocyte nuclear factor alpha (HNF4A), a promising candidate for attenuation of liver fibrosis.^49,50^ This LNP could successfully induce the production of HNF4A in fibrotic murine or human hepatocytes and strongly inhibit fibrogenesis in four kinds of liver fibrosis mouse models after intravenous injection. This research work broadens the application of mRNA LNP in liver diseases.

Liver cancer is a highly malignant tumor that is difficult to cure, and patients are often diagnosed at an advanced stage.^51^ Surgical resection, chemotherapy, and radiation therapy are by far the most common treatments for liver cancer.^52^ The rapid development of mRNA drugs provides new possibilities for the treatment of liver cancer. In one study, Liu and co-workers developed an LNP to deliver mRNA encoded with a single-domain antibody that could bind and neutralize CCL2 and CCL5 (BisCCL2/5i).^53^ CCL2 and CCL5 are two main cytokines that may induce the polarization of tumor associate macrophages (TAMs) to the M2-phenotype and mediate the immune escape of tumor cells.^54^ Therefore, repolarizing TAMs from the M2 phenotype to the M1 phenotype to reconstruct the antitumor microenvironment is crucial for tumor immunotherapy.^55^ They used the formulation of Onpattro to prepare the liver-targeted LNP and deliver the BisCCL2/5i mRNA to the liver. It was proven that bispecific antibodies could be expressed in the liver to polarize the TAMs into antitumor M1-phenotype. Together with anti-PD1 antibody therapy, this BisCCL2/5i mRNA-loaded LNP significantly extended the survival of mice bearing primary liver tumors or liver metastasis.

The loss-of-function mutation of the TP53 tumor suppressive gene is considered to be one of the main causes of carcinogenesis.^56^ It is reported that around 36% of hepatocellular carcinomas (HCCs) and 68% of non-small cell lung cancers (NSCLCs) were with TP53 gene mutation.^57^ Restoration of TP53 gene function may be a strategy for cancer therapy.^58^ Shi’s group recently reported a redox-responsive LNP loaded with p53-encoding mRNA.^59^ They investigated the therapeutic effect of these nanoparticles in p53-deficient Hep3B HCC and H1299 NSCLC. Strong apoptosis was observed both in *in vitro* cell experiments and *in vivo* animal models. Further investigation revealed the p53 restoration will increase the sensitivity of tumor cells to mTOR inhibitor, and combination therapy with p53-mRNA NPs and mTOR inhibitor will maximum the antitumor effect.

The abundant extracellular matrix (ECM) in the tumor microenvironment greatly restricts drug delivery to deep tumor cells, which usually leads to treatment failure.^60^ The activation of focal adhesion kinase (FAK) involves in the formation of dense ECM.^61^ To solve this problem and enhance the delivery efficiency of LNP to the tumor, Zhang et al. prepared an LNP loaded with Cas9 mRNA, PD-L1 sgRNA, and FAK siRNA.^62^ The cationic component used in this LNP is 5A2-SC8, which is an ionizable amino lipid dendrimer that facilitates the endosome escape of LNP. This LNP could not only inhibit xenograft tumor growth *in vivo* but also enhance gene editing in a liver cancer mouse model, demonstrating the reconstruction of tumor ECM together with immune checkpoint blockade by one LNP is a good choice for cancer immunotherapy.

In another study, Huang et al. use a novel LNP to deliver mRNA encoded with bispecific T-cell engaging (BiTE) antibody B7H3-CD3, which could bind with B7H3 receptors on tumor cells and CD3 receptors on T cells simultaneously, thereby inducing the recognition of tumor cells by T cells and antitumor efficacy.^63^ The LNP was composed of DMG-PEG, DSPC, cholesterol, and an ionizable cationic lipid IC8, with a size of 118 nm and a surface charge of 10 mV. After intravenous injection, this BiTE mRNA-loaded LNP will mainly accumulate in the liver and spleen and induce the activation of T cells specific to B7H3-positive tumor cells. This LNP showed a durable therapeutic effect against hematologic malignancies and melanoma.

Liver-targeted LNP could also be used for hereditary disease,^64^ including hemophilia and acute intermittent porphyria. Recent research revealed that the negative regulator antithrombin (AT) can be used as a target for the treatment of hemophilia A and B.^65,66^ Selective inhibition of AT by using RNA interference drug Fitusiran achieved the restoration of coagulation system balance.^67^ However, Fitusiran is a short-term drug that needs to be repeatedly injected.^68^ To find long-acting drugs for the treatment of hemophilia, Han et al. made an LNP loading with Cas9 mRNA and mouse AT-targeted sgRNA, to edit the AT gene and inhibit the activity of AT.^69^ This LNP carrier is composed of ionizable lipid, DOPE, cholesterol, and PEG lipid at the molar ratio of 26.5:20:52:1.5. Intravenous administration of this LNP resulted in the accumulation in the liver and long-term therapeutic effects on hemophilia A and B in mouse models. At the same time, the LNP does not cause hepatotoxicity and off-target toxicity. These results showed the LNP is safe and effective for hemophilia treatment.

Acute intermittent porphyria is a metabolic disease that is caused by the malfunction of porphobilinogen deaminase (PBGD), a key enzyme in the heme biosynthesis pathway.^70^ Fontanella’s group developed the LNP delivery system to deliver human PBGD (hPBGD) mRNA to hepatocytes, to reconstruct the expression of PBGD and keep it at a normal level.^71^ The results revealed that hPBGD could be expressed by mouse hepatocytes after the LNP was intravenously injected, which normalizes the excretion of porphyrin precursor by urine.

### 3.2 Spleen or Lung-Targeted LNP

**Figure 3:**
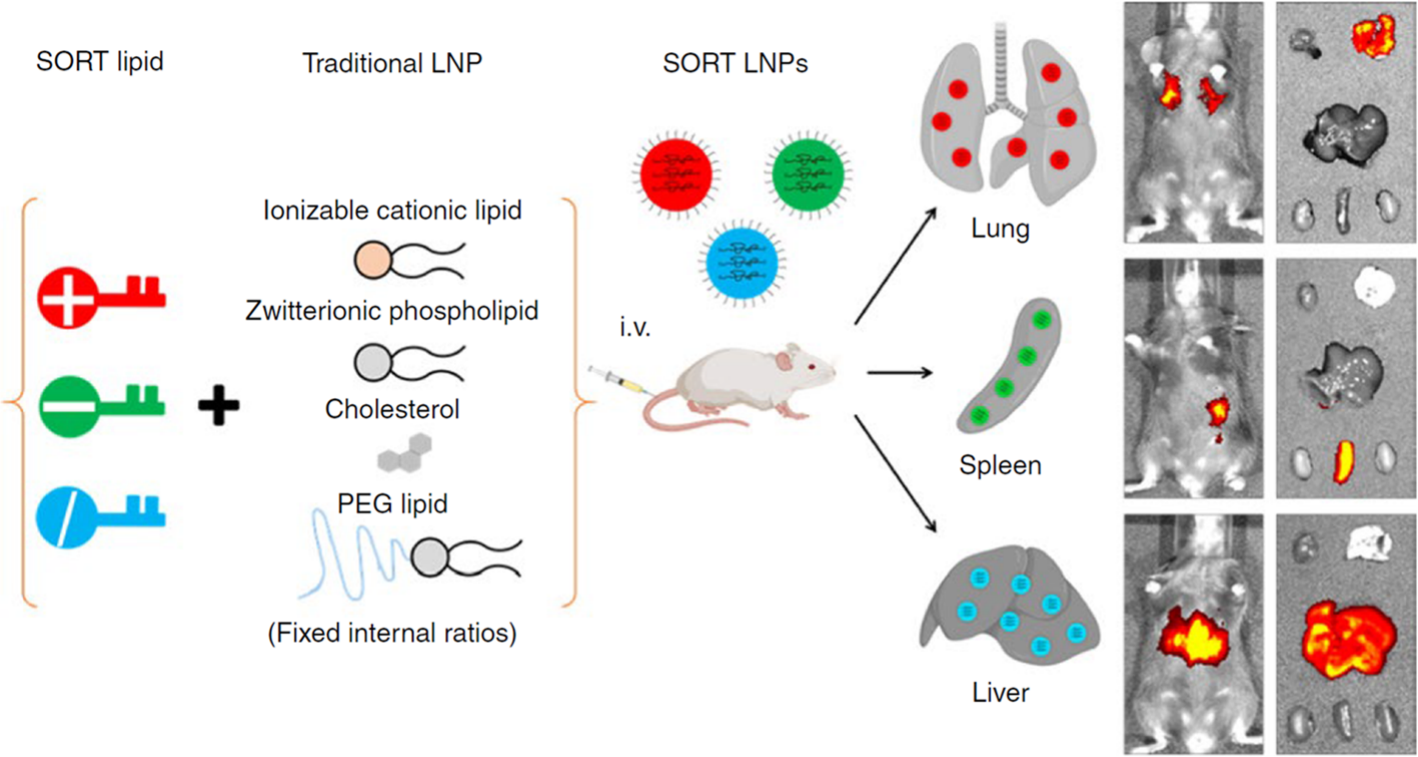
Design of selective organ targeting (SORT) nanoparticles and their organ-specific targeting delivery after intravenous injection. Traditional four-component LNP with fixed ratios is proved to be mainly delivered to the liver, which is caused by the interactions between adsorbed ApoE on LNP and LDL receptors on hepatocytes. To realize organ-specific drug delivery, the fifth component SORT lipid (DOTAP, 18PA, DODAP) was added to the prescription of LNP. By changing the SORT lipid with different charges or adjusting the percent of SORT lipid, organ-specific systemic delivery of LNP can be realized. Briefly, cationic lipid (DOTAP), anionic lipid (18PA), and ionizable lipid (DODAP) help LNP target to lung, spleen, and liver, respectively. Reprinted with permission from ref (72). Copyright 2020 Springer Nature.

The targeting ability of LNP to different organs can be controlled by adjusting the proportions of lipids in LNP. Recently, Siegwart groups found by changing the component and composition of LNP could realize the selective organ targeting ability, and they named this passive targeting LNP as SORT nanoparticles (Figure 3).^72^ With the advancement of organic synthesis, a lot of ionizable cationic and anionic lipids are commercially available. Adding the fifth lipid in the existing prescription of traditional four-component LNP and adjusting the proportion of this lipid could realize the selective targeting ability to different organs, including the liver, lung, and spleen. After *in vivo* screening, they found that the traditional four-component LNP will accumulate mainly in the liver and partially in the spleen, while the incorporation of the fifth lipid will change the distribution, and this effect was related to the proportion of the fifth lipid. When using cationic lipid as the fifth component, the distribution of LNP to the liver decreases when the proportion of the fifth lipid increases. As for the distribution of LNP to the lung and spleen, when the proportion of cationic lipids is higher than 50%, more than 90% of LNP is accumulated in the lung. While if the anionic lipid was chosen as the fifth lipid, the amount of LNP distributed to the liver decreased when the proportion of anionic lipid increased, and almost no LNP distributed to the lung. For the distribution of LNP to the spleen, the maximum distribution to the spleen was with the use of around 30% anionic lipid. If the fifth lipid was ionizable cationic lipid, the LNP distributed to the liver will increase and then decrease with the proportion of the fifth lipid increased. The 20% addition of ionizable cationic lipid realized the maximum distribution to the liver. Together, the incorporation of the fifth lipid will greatly affect the biodistribution of LNP to main organs, and 50% cationic SORT lipid, 30% anionic SORT lipid, and 20% ionizable cationic SORT lipid will facilitate the distribution of LNP to lung, spleen, and liver, respectively. Another work accomplished by Whitehead and co-workers got a similar conclusion. Further investigation by Siegwart revealed that this selective organ targeting effect was related to the categories of serum proteins that bind with LNP after intravenous injection.^73^

### 3.3 Bone-Targeted LNP

**Figure 4:**
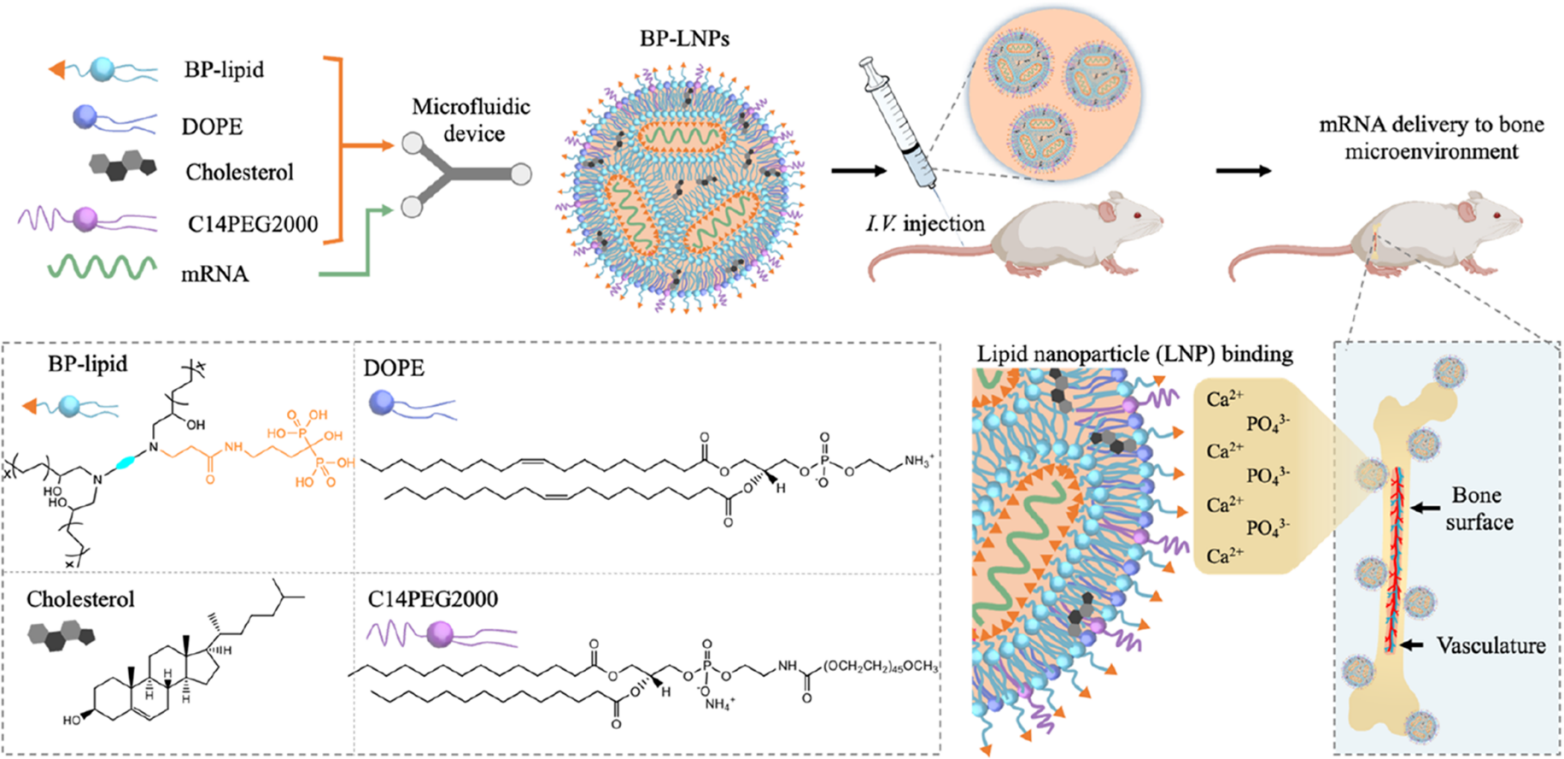
Design of the bisphosphonate lipid (BP)-functionalized LNP targeting to bone microenvironment after systemic administration. BP contains two phosphate groups, which chelate calcium ions in the bone microenvironment and help the LNP delivered to the bone microenvironment after intravenous injection. This BP-LNP contained four lipids (BP-lipid, DOPE, cholesterol, and C_14_PEG_2000_), and was fabricated by a microfluidic device. Reprinted with permission from ref (76). Copyright 2022 American Chemical Society.

The incidence of skeletal diseases and bone abnormalities increased in recent years, and there is an unmet medical need for new biomaterials that could target the bone microenvironment.^74^ It is reported that siRNA-loaded LNPs could be systemically delivered into bone marrow, while passive diffusion is still a challenge for bone-targeted drug delivery.^75^ Inspired by the fact that ligand substitution can realize targeted delivery by LNP. Mitchell and co-workers prepared a series of lipids with bisphosphonates (BP) head groups, which could strongly bind with calcium ions of hydroxyapatite on bone by chelation and realize the long-time retention in the bone microenvironment (Figure 4).^76^ They prepared the luciferase mRNA-loaded LNP by using different kinds of BP lipids and screened them by cell experiment. Results showed the 490BP-C14 LNP was best for transfection. Furthermore, the bone morphogenetic protein-2 (BMP-2) encoded mRNA was loaded into this LNP, which showed excellent bone microenvironment targeting ability and high expression of BMP-2 in bone tissues after intravenous injection.

## 4 Cell-Specific LNP by Systemic Administration

To get a more specific mRNA delivery system, cell-targeted LNP was developed that was designed to be taken up by specific cells, resulting in protein expression in these cells. Typically, this cell-specific uptake was mediated by ligand–receptor interactions, while recent research found this effect can be realized by changing the lipid structure in LNP.

### 4.1 Leukocyte-Targeted LNP

**Figure 5:**
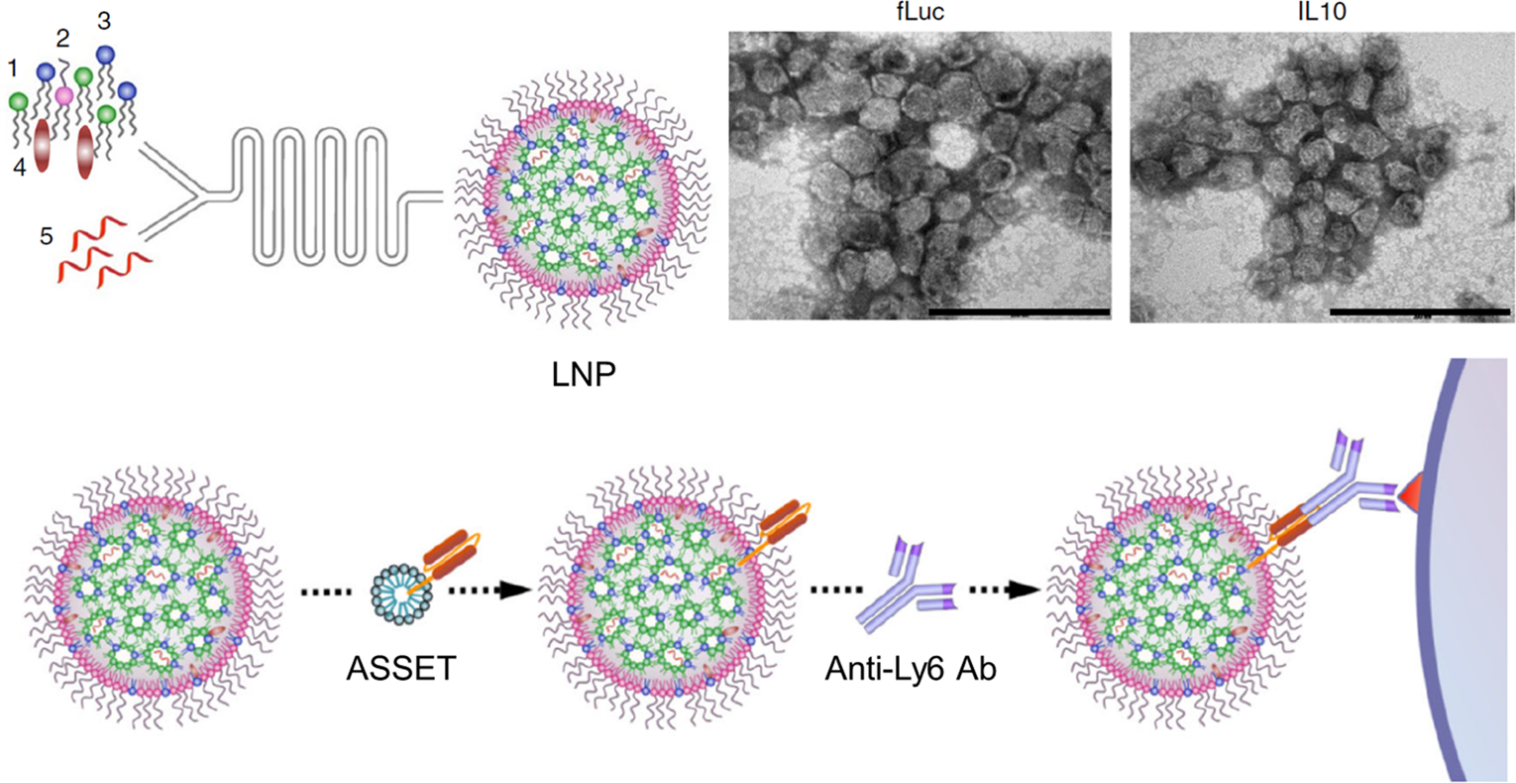
Preparation of leukocyte-specific LNP. Unmodified LNP was first prepared by microfluidic mixing of lipids (1–4) and therapeutic mRNA (5). To anchor a leukocyte-specific ligand to LNP, ASSET micelle was mixed with LNP, which allows the further conjugation of anti-Ly6 antibody to the surface of LNP. ASSET is a modular targeting platform that bridges the LNP and specific antibodies to realize the construction of antibody-modified LNP. Adapted with permission under a Creative Commons CC BY License from ref (77). Copyright 2018 Springer Nature.

To realize a cell-specific mRNA therapy for inflammatory bowel disease (IBD), the Peer group prepared an antibody-modified LNP for targeted delivery of IL-10 mRNA to Ly6c^+^ inflammatory leukocytes (Figure 5).^77^ IBD is an incurable autoimmune disease, and the chronic inflammation greatly affects the patient’s life quality.^78^ Ly6c^+^ leukocytes are vital to inflammation disease, which could be used as a target for the treatment of IBD.^79^ It is reported that the IBD could be inhibited by immunosuppressive cytokine IL-10.^80^ To induce the long-term production of IL-10, LNP loaded with IL-10 mRNA was first prepared, followed by incubation with ASSET micelle at 4 °C for 48 h and incubation with anti-Ly6c mAbs for 30 min. Here, the ASSET (Anchored Secondary scFv Enabling Targeting) is an original modular targeting platform created by the Peer group, which could bridge LNP with targeting antibodies in mild conditions. After intravenous injection into dextran sodium sulfate-induced colitis mice, this surface-modified LNP actively targets Ly6c^+^ leukocytes and induces the production of IL-10, thereby significantly inhibiting inflammation in the colon.

### 4.2 T Cell-Targeted LNP

**Figure 6:**
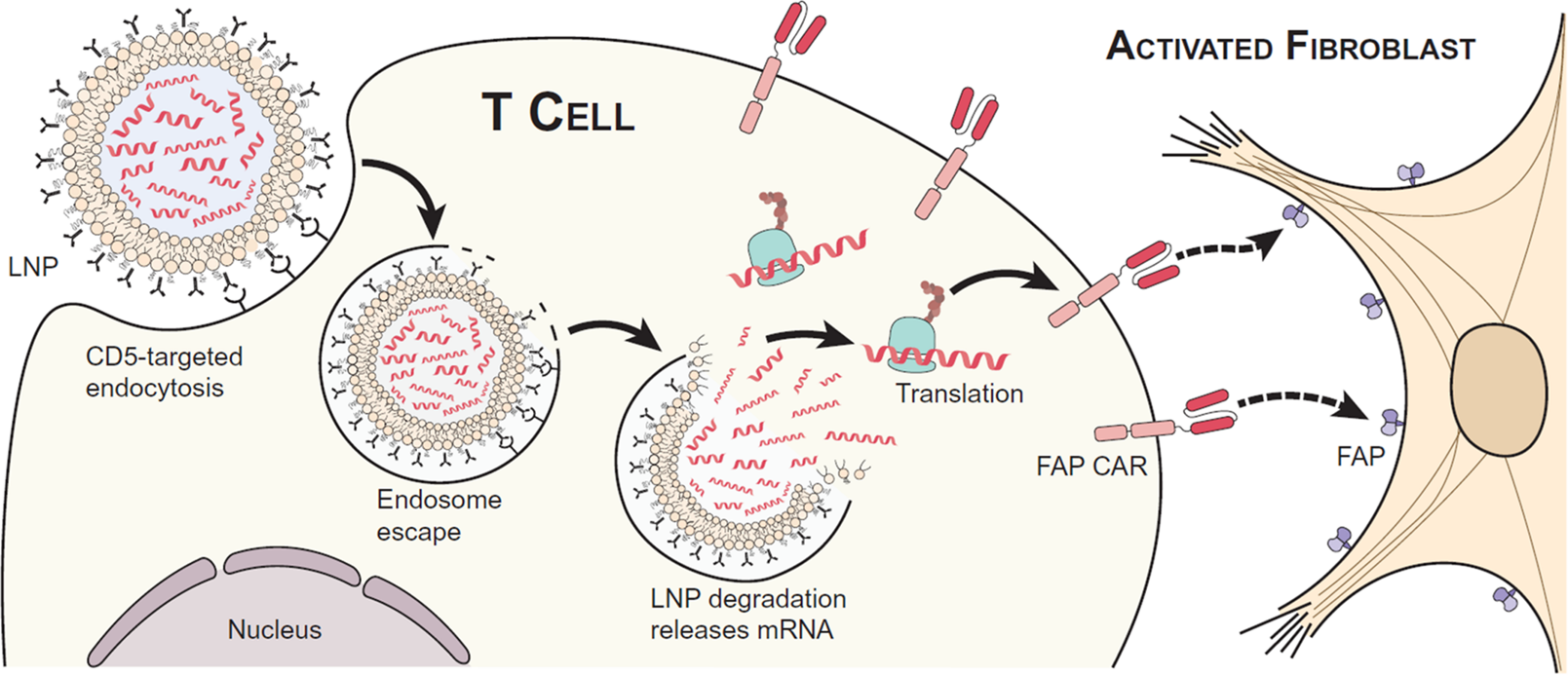
Anti-CD5 antibody modified LNP for *in vivo* construction of CAR T cells specific to fibroblast activation protein (FAP), to trogocytose FAP from activated cardiac fibroblasts and improve cardiac function. Overexpressed FAP is considered a sign of cardiac injury. To reverse this injury, *in vivo* construction of FAPCAR T cell was reported, which was mediated by T cell-specific mRNA delivery. FAP CAR protein encoded by mRNA could be anchored to T cells to exert the anti-FAP activity. The T cell-targeted delivery of mRNA was realized by anti-CD5 antibody-modified LNP, in which the anti-CD5 antibody was chemically conjugated to the LNP surface after the LNP was prepared. Reprinted with permission from ref (83). Copyright 2022 The American Association for the Advancement of Science.

Cardiac fibrosis is caused by the excessive extracellular matrix produced by cardiac fibroblasts.^81^ Limiting fibrotic progression by antifibrotic therapeutics is not satisfactory for the treatment of cardiac fibrosis.^82^ Recently, Rurik et al. developed a CAR-T therapy to remodel fibrosis, which was realized by *in vivo* targeted delivery of mRNA to T cells (Figure 6).^83^ Fibroblast activation protein CAR (FAP-CAR) was encoded into an mRNA, which was then loaded into an anti-CD5 antibody modified LNP to realize the specifical targeting to CD5^+^ T cells. The FAP-CAR expressed at the surface of T cells, which specifically recognized the FAP-positive cells and induced cell death, leading to the alleviation of cardiac fibrosis. They also studied the therapeutic effect in the angiotensin II/phenylephrine-induced mouse cardiac injury model. The systemic administration of CD5-targeted LNP loaded with FAP-CAR mRNA significantly reduced fibrosis and improved cardiac function. This is a great success of mRNA-loaded LNP in the treatment of heart disease.

### 4.3 Kupffer Cells and Liver Endothelial Cells Targeted LNP

Kupffer cells play an important role in liver inflammation and immune tolerance, and they clear particles mainly through phagocytosis. It is reported that increasing the size of LNPs and surface modification of LNP with hydrophobic molecules could enhance the cellular uptake and promote immune regulation of Kupffer cells.^84^ Liver sinusoidal endothelial cells (LSECs) reside in the liver sinusoids and are responsible for blood filtration, metabolism regulation, antigen presentation, and lipid metabolism.^85^ To realize the Kupffer cell or LSECs specific mRNA delivery, Dahlem group applied many different kinds of cholesterol in the prescription of LNP, and found the structure of cholesterol has a great influence on the targeting ability of LNP.^86^ They screened LNP formulated with different oxidized cholesterol with fast identification of nanoparticle delivery (FIND) system, and conclude that LNPs containing oxidized cholesterol will deliver mRNA to cells in the liver microenvironment, including Kupffer cells and liver endothelial cells, which is different from the traditional LNP that mainly target to hepatocyte. Considering the important role of Kupffer cells and LSECs in antigen presentation and anti-inflammation effects, this LNP could be further used for immunotherapy.

Pattipeiluhu et al. prepared a scavenger receptor LNPs (srLNPs) that could specifically target sinusoidal endothelial cells.^87^ Liver sinusoidal endothelial cells (LSECs) and Kupffer cells (KCs) are the primary cell types of the hepatic blood vessel and sinusoids, which are responsible for liver homeostasis. Moreover, LSECs can function as antigen-presenting cells for regulating adaptive immunity and immunotolerance, considered a good target for immunotherapy. Based on the previous studies, anionic nanoparticles can be taken up by SECs, which are mediated by the stabilin receptors. Here, they prepared the srLNPs by referring to the composition of Onpattro. The only difference is the zwitterionic lipid DSPC was replaced by anionic lipid DSPG. After being injected into zebrafish, the srLNPs will accumulate into SECs, which was mediated by stabilin-1 and -2 mediated cellar uptake by SECs.

Recently, our group exploited an LSEC-targeted LNP for the treatment of peanut-induced food allergy.^85^ The targeting ability to LSEC was realized by modification of mannose on LNP, which comes from the incorporation of mannose containing lipid DSPE-PEG_2000_-Mannose in the prescription of it. The mRNA used in the delivery system is encoded with dominant epitope peptides from a major peanut allergen, *Arachis hypogaea* protein 2 (Ara h2). The size of this mannose-modified LNP was around 150 nm, with neutralized surface charge. After intravenous injection, the LNP will mainly accumulate in the liver. The cellular uptake of mannose-modified LNP by LSEC was proved to be higher than unmodified LNP. For the therapeutic effect, the mannose-modified LNP was proved to induce a tolerogenic effect in peanut induced food allergic mice, detailed by the suppressed anaphylactic response to peanut allergen, accompanied by decreased IgE production, mast cell protease 1 release and Th2 cytokine release. This LNP is a promising platform for allergic disorders and autoimmune diseases.

Interestingly, the targeting ability of LNP to different liver cells was also influenced by the proportion of PEG lipids.^88^ With the increase of PEG lipid proportion from 1.0% to 3.0%, more LNPs are delivered to hepatocytes, together with the decreased delivery to Kupffer cells and liver sinusoidal endothelial cells. Moreover, the LNP will specifically target to LSECs if the PEG lipid was partially replaced by mannose modified lipid, which demonstrates that the cell-specific delivery of mRNA could be realized by changing the proportion and structure of the PEG lipid.

## 5 Conclusion and Perspectives

LNP-based mRNA therapy is a promising nucleic acid therapy that can target many diseases that are hardly targeted by small molecules. The success of the mRNA vaccine in the COVID-19 pandemic boosts the development of mRNA-loaded LNP for the treatment of many different diseases, including infections, cancer, and genetic disease. The physical properties of LNP, including particle size, morphology, and surface properties, are greatly influenced by lipid structure and composition, and these physical properties will affect the efficacy, safety, and drug distribution of LNP. In this review, we introduce the site-specific LNP for mRNA delivery from three aspects, including LNP that was locally injected, organ-targeted LNP after intravenous injection, and cell-targeted LNP after intravenous injection.

Local administration of LNP allows the direct interactions between LNP and target tissues or organs. Many routes of administration have been proven to be effective for mRNA delivery by LNP, including oral administration for the treatment of IBD, inhalation for the treatment of H1N1 infection, etc. The advantage of local administration is the high delivery efficiency and low systemic toxicity. However, local injection is difficult to achieve for some diseases such as brain disorders and metastatic tumors.

In addition to local administration, intravenous injection is widely used for LNP drugs. The first FDA-approved LNP drug Onpattro mainly accumulate in the liver after intravenous injection, which was mediated by the interaction of adsorped ApoE on LNP and LDLR on hepatocytes. To achieve specific delivery of LNP to other organs or cells, a large number of strategies were used, including (1) changing the structure of functionalized lipids, including ionizable lipids, cholesterol, and PEGylated lipids; (2) changing the ratio of different lipids in LNP; (3) surface modification after the preparation of LNP. All of these change the distribution of LNPs after intravenous injection by increasing the nonspecific or specific interactions with different target organs or cells. There are still some challenges and problems that remain unsolved.^89^ For the first strategy, the structure change of different lipids may introduce unexpected toxicities. Although the Onpattro has been proven to be safe, the safety of new lipids was not guaranteed. It is reported the ionizable cationic lipid with lower p*K*_a_ may cause excessive disruption of the lysosome, leading to lysosomal dysfunction and cell death. For the second strategy, the change in lipid ratio may influence the physiochemical properties of LNP, including size, charge, and stability. This strategy needs more studies to explore their long-term toxicity. For the third one, the grafting rate of different antibodies on the LNP is not investigated in these research works, which is an important parameter because it influences the targeting efficiency and off-target toxicity directly.

There are also some challenges regarding the mRNA and nanoparticles themselves.^90^ It is reported that the protein expression from mRNA is transient, which required the repeated injection of mRNA-loaded LNP. However, the repeated injection may generate immune responses such as neutralizing antibodies against PEGs, which in turn reduces the drug efficacy. Therefore, it is important to find disease-specific mRNA to increase the drug efficacy and decrease the injection frequency and related immunogenicity. It is also reported that intravenous injection nanoparticles can lead to hypersensitivity reactions, which is a type of pseudoallergy and was explained by unexpected complement activation.^91^ Further development of LNP should rule out or decrease the chance of this allergic reaction.

Despite the challenges in developing site-specific mRNA therapeutics, we are optimistic that advancements in chemical synthesis and nanotechnology will lead to the launch of increasingly effective treatments. This holds great promise for improving health outcomes and expanding access to effective therapies for a wider population.
